# Associations between dual use of combustible cigarettes and electronic nicotine delivery systems and allergic rhinitis: An analysis of KNHANES data^☆^

**DOI:** 10.1016/j.waojou.2026.101423

**Published:** 2026-07-02

**Authors:** Dong Ju Yoon, Jiyoon Yeo, Ye-Seul Lee, Chunhoo Cheon, Yoon Jae Lee, In-Hyuk Ha

**Affiliations:** a Jaseng Hospital of Korean Medicine, 536 Gangnam-daero, Gangnam-gu, Seoul 06110, Republic of Korea; b Jaseng Spine and Joint Research Institute, Jaseng Medical Foundation, 2F, 540 Gangnam-daero, Gang-nam-gu, Seoul 06110, Republic of Korea; c Department of Preventive Medicine, College of Korean Medicine, Kyung Hee University, Seoul, Republic of Korea

**Keywords:** Allergic rhinitis, Combustible cigarettes, Electronic nicotine delivery systems, Korean national health and Nutrition examination survey

## Abstract

**Background:**

The dual use of combustible cigarettes (CC) and electronic nicotine delivery systems (ENDS) is a rapidly growing public health concern, yet its synergistic impact on allergic rhinitis (AR) remains poorly understood. Previous studies have reported inconsistent associations between smoking and AR, often failing to account for different tobacco product combinations and the temporal sequence of smoking initiation.

**Objective:**

To analyze the association between diverse smoking behaviors—including exclusive CC use, exclusive ENDS use, and dual use—and the odds of AR diagnosis among Korean adults.

**Methods:**

We analyzed data from 18,781 adults from the 2019–2022 KNHANES. Multivariable survey-weighted logistic regression estimated adjusted odds ratios (AOR) across 5 patterns: never, former, exclusive CC, exclusive ENDS, and dual users. Sensitivity analyses were conducted to establish temporal precedence by restricting the sample to those who initiated smoking prior to their AR diagnosis. Further stratified analyses were performed based on smoking intensity (Cigarettes Per Day [CPD]).

**Results:**

The overall weighted prevalence of AR was 17.77%. In the primary model, exclusive CC smokers showed lower odds of AR (AOR = 0.691; 95% CI = 0.562–0.850), which decreased further to 0.519 (p < 0.001) upon temporal validation. While the primary model showed higher odds for former smokers (AOR = 1.193), the validated subset revealed a significant inverse association (AOR = 0.836, p = 0.018), identifying a “sick quitter” effect. Conversely, dual use of CC and vapes was significantly associated with higher AR odds (AOR = 1.543; 95% CI = 1.067–2.233, p = 0.021). This risk peaked among moderate smokers using vapes (AOR = 2.222, p = 0.029) and heavy smokers using heated tobacco products (AOR = 2.374; 95% CI = 1.059–5.322, p = 0.036).

**Conclusion:**

Our findings reveal a distinct contrast between exclusive smoking and dual use. While exclusive CC smoking exhibits an inverse association likely driven by nicotine-mediated symptom masking, dual use presents a unique synergistic risk. This suggests that combined exposure to multiple tobacco products may exacerbate mucosal inflammation and barrier dysfunction, overriding any suppressive effects of nicotine.

## Introduction

Allergic rhinitis (AR) is the most prevalent airway disease globally, affecting an estimated 500 million individuals with a chronic inflammatory burden that continues to escalate across diverse populations.^1^^,^^2^ AR is not just nasal symptoms resulting from problems in the nasal mucosa; it is closely associated with lower airway diseases such as asthma, and when the condition becomes chronic, it adversely affects the overall quality of life, including sleep disorders, chronic fatigue, and poor concentration.^3^

The etiology for the development of AR involves complex interactions of genetic factors and a variety of environmental factors, such as air pollution, changes in indoor environment, smoking, and changes in diet. Of these factors, smoking is one of the major risk factors of AR that directly affects the mucosa across the respiratory system, and various chemicals contained in the cigarette smoke and e-cigarette aerosol may cause or worsen AR symptoms by altering the immune responses and causing inflammatory responses.^4^

However, previous studies that have examined the relationship between smoking and AR have reported inconsistent findings. Some studies reported that smoking suppresses allergic immune responses, thereby reducing the onset of AR. Others argued that smoking enhances pro-inflammatory responses in the nasal mucosa, thus exacerbating AR symptoms. These mixed results from previous studies are considered to be related to recent changes in the patterns of smoking behavior;^5^^,^^6^ specifically, the use of traditional combustible cigarettes (CC) has dropped in the 21st century, while the new electronic nicotine delivery systems (ENDS), such as e-cigarettes, have emerged.

Since the late 2010s, not only electronic vapor products (Vapes) but also heat-not-burn products (HnB) have become popular among the public, and the marketing strategy of "less harmful than traditional cigarettes because they do not involve combustion" succeeded in attracting new consumer groups, including youth and non-smokers, as well as the existing smokers of CC.^7^

Recently, "dual use," a new pattern of smoking behavior has emerged. Some smokers, instead of replacing CC with ENDS completely, use vape or HnB along with their existing habit of smoking CC. Indeed, a significant proportion of smokers incorporate ENDS into their habits as a perceived cessation aid; many initiate electronic cigarette use as a strategic transition to reduce or quit combustible smoking. However, longitudinal evidence suggests that such efforts frequently result in sustained dual use, effectively diversifying nicotine delivery patterns and prolonging tobacco exposure instead of facilitating complete cessation.^8^^,^^9^ Nevertheless, little is known about the health implications of such dual use, particularly the long-term effects on respiratory diseases such as AR.^10^

This study aimed to analyze the correlation between the type of smoking behavior and OR for AR among Korean adults, using data from the Korean National Health and Nutrition Examination Survey (KNHANES) from 2019 to 2022. Specifically, to examine the differences by smoking type in more detail, which has not been investigated previously, the smoking types were classified as: 1) use of CC only, 2) use of ENDS only, and 3) dual use of CC and ENDS for comparison.

## Data and analysis methods

### Data and samples

In this study, data from the KNHANES was used, which is a nationwide, cross-sectional survey based on a multistage probability sampling design, conducted annually to produce health statistics for the Korean population.^11^ This study used integrated data from 4 years, 2019–2022, which included questions on the use of both HnB and Vapes.

**Fig. 1:**
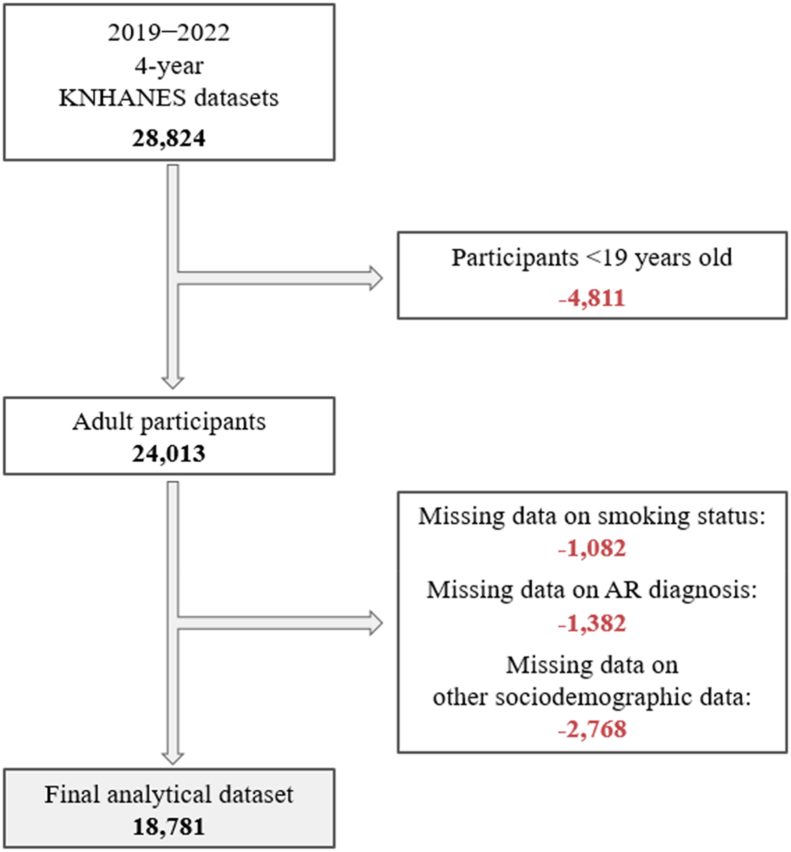
Data flowchart.

The sample selection process is detailed in Fig. 1. The initial 4-year dataset included 28,824 participants; 4811 participants from minors aged—under 19 years—which is the legal smoking age in South Korea, were excluded from the total participants. From the remaining 24,013 adult participants, we sequentially excluded participants with missing data on key variables.

We excluded 1082 participants with no response to the current smoking status questions (our main independent variable). Subsequently, 1382 participants with missing responses on whether they had been diagnosed with allergic rhinitis by a physician (our dependent variable) were also removed.

Finally, to conduct a complete case analysis for our adjusted models, we excluded participants with missing data on any of the sociodemographic covariates. A total of 2768 individuals were removed due to missing data on at least 1 of the 8 covariates used in the analysis (occupation, marital status, obesity, education, income, stress, residential type, and drinking status). The vast majority of these exclusions were due to missing responses on drinking status, which accounted for 2395 missing values.

After applying these exclusion criteria, a total of 18,781 participants were included in the final analytical dataset.

### Variables

#### Independent variables: smoking patterns and product use

The independent variables used in this study focused on the patterns of combustible cigarettes (CC) and electronic nicotine delivery system (ENDS) use, specifically heated tobacco products (HnB) and liquid-type vapes.

In the analysis of the general population, participants were classified into 4 primary categories based on their smoking status: never-smokers were defined as individuals with no lifetime experience of either CC or ENDS use; former smokers included those with a lifetime history of CC or ENDS use but who were not currently using either product; current CC smokers were defined as those smoking CC daily or occasionally; and current ENDS users encompassed individuals currently using HnB (daily or occasionally) or vapes (lifetime experience with use within the past month).

In the subsequent stratified analysis conducted among current smokers, the population was further categorized to evaluate the specific associations of dual-use patterns. In this specific framework, exclusive CC smokers (CC-only)—defined as current CC smokers who do not currently use any form of ENDS—served as the reference group. Dual use was then categorized by the concurrent use of CC with HnB (current daily or occasional use) or vapes (lifetime experience and use within the past month).

Although HnB and vapes are both classified as ENDS, they were analyzed separately because of their distinct aerosol generation mechanisms and chemical.

#### Dependent variable: having AR

The dependent variable in this study was the lifetime diagnosis of AR. The definition was based on the KNHANES questionnaire item asking, "Have you ever in your life been diagnosed with allergic rhinitis by a doctor?"

To align with our research objective of examining associations with chronic risk factors, we defined AR based on lifetime physician diagnosis. This method, consistent with prior NHANES research, is more appropriate for analyzing cumulative effects than estimating point prevalence.^12^

Participants who answered "Yes" were defined as patients with AR, and those who answered "No" were classified as non-AR patients.

#### Other covariates related to sociodemographic characteristics

The following sociodemographic and health-related variables were used as covariates for the regression analysis in this study: sex, age, marital status, occupational category, education, income quintiles, alcohol consumption, weight status (obesity level), stress level, residence type, living children, and the year of data collection (investigation year).

The age category was divided into 7 ten-year groups, from those in their 20s–70s and above. The marital status category was classified into 3 groups: single, married, and divorced/widowed/separated. The occupation category was classified into 5 groups: unemployed; managers, professionals, and office workers; service and sales workers; skilled agricultural, forestry, and fishery workers; and a combined group of craft and related trades workers, plant and machine operators and assemblers, and elementary occupations. The education category was classified into 3 groups: middle school graduate or below, high school graduate, and college graduate or above. The income category was classified into quintiles using the official household income quintile variable provided in the KNHANES data, which is calculated and provided annually by the KDCA.

The alcohol consumption category was classified into 3 groups: non-drinker, moderate drinker (1–3 glasses for women, 3–5 glasses/time for men), and heavy drinker (4 or more glasses/time for women, 5 or more glasses for men). The weight status category was classified into 4 groups: normal, underweight, pre-obesity, and obesity. The stress level category was classified into 3 groups: almost none, moderate, and high. The type of residence was classified into 2 groups: residence in apartment housing and others. The samples were also classified into the status of living with children aged under 13 years (yes/no). Finally, the year of data collection (investigation year) was used as another covariate to adjust for differences from the influence of time-related factors.

### Statistical analysis

For statistical analysis in this study, the characteristics of the KNHANES data (the survey was based on a complex sampling design) were considered.

For analysis on the differences in the characteristics of participants between those diagnosed with AR and those without the AR diagnosis, all independent variables and covariates were defined using categorical values and the Rao-Scott Chi-square test was performed. Further, to examine the associations between smoking behaviors and AR, multivariable survey-weighted logistic regression analyses were performed in a hierarchical manner.

First, we evaluated the associations of former and exclusive CC smoking within the general population relative to never-smokers. Subsequently, we analyzed the associations of exclusive ENDS use—including both former and current users—specifically among individuals with no lifetime history of CC smoking. A comprehensive model was then constructed to categorize the total population into 5 groups—never-smokers, former smokers, exclusive CC smokers, exclusive ENDS users, and dual users—to evaluate the independent and combined associations of these products while accounting for past exposure. Adjusted models included sex, age, marital status, occupation, education, income, drinking status, weight status, stress level, type of residence, living with children under 13 years, and the investigation year as potential confounders.

Furthermore, to address the potential for reverse causality—specifically the "sick quitter" effect—we conducted a sensitivity analysis to establish temporal precedence between CC smoking initiation and AR diagnosis. This analysis was restricted to individuals whose CC initiation preceded or coincided with their AR diagnosis (initiation age ≤ AR diagnosis age), while those who initiated smoking after their diagnosis (initiation age > AR diagnosis age) were excluded. This methodological step ensured that the CC exposure chronologically preceded the clinical onset of AR, providing a more robust estimate of the associations.

In addition, we hypothesized that the association between dual use and AR might be modified by the baseline intensity of CC smoking (ie, nicotine dependence). The patterns and motivations for ENDS use may differ between heavy and light CC smokers, potentially leading to different health risks. First, the entire smoker population was analyzed and then divided into 3 groups based on CPD: light (<10 cigarettes/day), moderate (10–19 cigarettes/day), and heavy (≥20 cigarettes/day). In these stratified models, exclusive CC-only smokers served as the reference group to evaluate the additional association of ENDS use among active smokers.

All regression analyses were performed using the “survey” package in R version 4.4.0, and a significance level of p < 0.05 was used for two-tailed tests.

## Results

### General characteristics of the participants

Table 1 presents the basic sociodemographic characteristics of study participants by presence/absence of AR. Statistically significant differences between groups were observed for all socioeconomic and demographic variables except the year of investigation. Of the 18,781 participants, 3068 (17.77%) reported that they were diagnosed with AR. The distribution of smoking types varied significantly according to AR status (p < 0.001). In the AR group, never-smokers accounted for 58.2%, while former smokers and CC-only users comprised 21.4% and 11.9%, respectively. The percentages of ENDS-only users (3.3%) and dual users (5.1%) were higher in the AR group compared to the group without AR diagnosis (2.8% and 3.8%, respectively)

**Table 1:** Basic characteristics of study participants by presence of allergic rhinitis.

	Total		Allergic rhinitis diagnosis						P-value∗
			No			Yes			
	n	% (C)	n	% (C)	% (R)	n	% (C)	% (R)	
**Overall**	18,781	100	15,713	100	82.23	3068	100	17.77	<0.001
**Smoking type**									<0.001
Never	10,536	56.10	8662	52.5	80.66	1874	58.2	19.34	
Former	4437	23.62	3779	23.9	83.79	658	21.4	16.21	
CC-only	2798	14.90	2471	17.1	86.87	327	11.9	13.13	
ENDS-only	432	2.30	342	2.8	79.5	90	3.3	20.5	
CC × ENDS	578	3.08	459	3.8	77.42	119	5.1	22.58	
**Sex**									<0.001
Male	8965	52.73	7765	54.4	84.83	1200	45.02	15.17	
Female	9816	47.26	7948	45.6	79.33	1868	54.98	20.67	
**Age**									<0.001
<30	2616	18.70	1954	16.86	74.1	662	27.26	25.9	
30–39	2715	17.57	2043	16.24	76.01	672	23.72	23.99	
40–49	3535	20.25	2876	20.04	81.39	659	21.21	18.61	
50–59	3597	20.02	3056	20.81	85.48	541	16.36	14.52	
60–69	3566	14.71	3211	16.12	90.15	355	8.15	9.85	
70+	2752	8.75	2573	9.93	93.3	179	3.3	6.7	
**Marital status**									<0.001
Single	3935	26.92	3020	25.01	76.39	915	35.77	23.61	
Married	12,527	63.83	10,657	65.22	84.03	1870	57.38	15.97	
Divorced/Widowed/Separated	2319	9.25	2036	9.77	86.84	283	6.85	13.16	
**Occupation**									<0.001
Unemployed	6904	33.74	5812	33.59	81.86	1092	34.45	18.14	
Managers, professionals, Clerks	5061	30.25	4019	29.09	79.08	1042	35.62	20.92	
Sales, service workers	2513	14.19	2043	13.89	80.48	470	15.59	19.52	
Agriculture, forestry, Fisheries	705	2.46	662	2.79	93.15	43	0.95	6.85	
Trades workers, laborers	3598	19.35	3177	20.64	87.7	421	13.4	12.3	
**Education**									<0.001
≤Middle school	4407	16.47	4065	18.33	91.47	342	7.91	8.53	
High school	6630	37.55	5481	37.38	81.87	1149	38.32	18.13	
≥College	7744	45.98	6167	44.29	79.22	1577	53.78	20.78	
**Income**									<0.001
Quintile 1	2283	9.42	2034	9.92	86.62	249	7.09	13.38	
Quintile 2	3239	14.85	2796	15.27	84.56	443	12.9	15.44	
Quintile 3	3957	21.70	3297	21.74	82.37	660	21.54	17.63	
Quintile 4	4518	26.05	3688	25.61	80.84	830	28.09	19.16	
Quintile 5	4784	27.98	3898	27.47	80.71	886	30.38	19.29	
**Drinking status**									0.026
Non-drinker	3985	18.57	3418	18.96	83.96	567	16.76	16.04	
Moderate drinker	9552	49.42	7936	48.96	81.46	1616	51.58	18.54	
Heavy drinker	5244	32.01	4359	32.08	82.42	885	31.66	17.58	
**Weight status**									<0.001
Normal	6919	36.10	5682	35.42	80.67	1237	39.28	19.33	
Underweight	769	4.33	616	4.12	78.15	153	5.33	21.85	
Pre-obesity	4335	22.68	3687	22.97	83.28	648	21.33	16.72	
Obesity	6758	36.89	5728	37.5	83.59	1030	34.06	16.41	
**Stress level**									<0.001
Almost none	2902	13.95	2568	14.66	86.39	334	10.69	13.61	
Moderate	10,898	58.18	9237	59.11	83.55	1661	53.86	16.45	
High	4981	27.87	3908	26.23	77.4	1073	35.45	22.6	
**Residence in apartment housing**									0.006
No	10,393	59.64	8550	59.03	81.4	1843	62.44	18.6	
Yes	8388	40.36	7163	40.97	83.47	1225	37.56	16.53	
**Living with children aged under 13 years**									<0.001
No	15,195	79.30	12,885	80.19	83.15	2310	75.2	16.85	
Yes	3586	20.70	2828	19.81	78.71	758	24.8	21.29	
**Investigation year**									0.101
2019	5197	24.90	4393	25.16	83.1	804	23.69	16.9	
2020	4724	24.80	3943	24.9	82.56	781	24.35	17.44	
2021	4596	25.33	3876	25.46	82.63	720	24.77	17.37	
2022	4264	24.96	3501	24.48	80.64	763	27.2	19.36	

Abbreviations: CC, combustible cigarettes; ENDS, electronic nicotine delivery systems. Note: Data are presented as unweighted n (weighted column %, weighted row %). ∗P-value from the Rao-Scott Chi-square test for the association between the presence of allergic rhinitis and demographic variables under the KNHANES survey design

From a clinical prevalence perspective (Row %), the absolute proportion of AR diagnosis within each smoking category showed a distinct pattern. While the overall weighted prevalence was 17.77%, the highest prevalence was observed among dual users (22.58%) and exclusive ENDS-only users (20.5%). In contrast, exclusive CC-only users exhibited a markedly lower prevalence of 13.13%. Never-smokers and former smokers showed intermediate prevalence rates of 19.34% and 16.21%, respectively.

Sex showed a significant association with the OR of AR, with women (54.98%) accounting for higher percentage than men (45.02%) in the AR group. Age was also associated with AR, with a higher odd ratio for AR observed among younger participants aged under 50 years. The percentage of single participants in the AR group was 35.77%, higher than in the non-AR group (25.01%). Furthermore, the percentage of those engaged in indoor occupations such as managers, professionals, and office workers (35.62%) was higher in the AR group. On the contrary, the percentage of those working with outdoor occupations, such as in agriculture and fishing, was higher in the non-AR group (2.79%).

The percentage of those who were middle school graduates or lower was higher in the non-AR group (18.33%), while the percentage of those who were college graduates or above was higher in the AR group (53.78%). The percentage of those in the income quantile 2 or below was higher in the non-AR group (25.19%) than in the AR group (19.99%). The non-AR group had a slightly higher percentage of respondent who were non-drinkers (18.96%), and the percentage of those who were pre-obese or obese was also higher (60.47%) in the non-AR group than in the AR group (55.39%). In addition, the percentage of those living in apartment housing was lower (37.56%) in the AR group than in the non-AR group (40.97%), and the percentage of those living with children aged under 13 years was higher (24.80%) in the AR group than in the non-AR group (19.81%).

### Distribution of smoking behavior types by cigarettes per day (CPD)

Table 2 presents the distribution of smoking behavior types by CPD for participants classified as smokers. Of the 3808 smokers, 70.05% (n = 2798) reported using CC only, 12.46% (n = 432) reported using ENDS only, and the remaining 17.49% (n = 578) were dual users of CC and ENDS (calculated as 100%–70.05% - 12.46%). Notably, although dual users represented a minority (17.49%) of the total smoking population, they constituted the majority (57.23%) of all ENDS users in our sample (n = 578 out of 1010 total ENDS users, including 432 exclusive and 578 dual users). This indicates that ENDS utilization in the Korean adult population is predominantly characterized by concurrent combustible cigarettes consumption rather than exclusive product substitution.

**Table 2:** Distribution of smoking behavior types by cigarettes per day.

Smoking Type	Cigarettes Per Day (CPD)												Row Total		
	ENDS only			Light (1 ≤ CPD <10)			Moderate (10 ≤ CPD <20)			Heavy (20 ≤ CPD)					
	N	% (R)	% (C)	N	% (R)	% (C)	N	% (R)	% (C)	N	% (R)	% (C)	N	% (R)	% (C)
CC-only	–	–	–	793	28.49	73.44	1180	43.06	82.32	825	28.46	84.00	2798	100.00	70.05
HnB	270	44.75	62.49	134	24.02	15.39	120	18.79	8.92	69	12.44	9.12	593	100.00	17.40
Vape	123	42.24	29.16	72	24.74	7.83	56	20.93	4.92	37	12.10	4.39	288	100.00	8.60
Both ENDS	39	26.35	8.35	27	23.02	3.35	45	35.67	3.84	18	14.96	2.49	129	100.00	3.95
**Column total**	432	12.46	100.00	1026	27.17	100.00	1401	36.64	100.00	949	23.73	100.00	3808	100.00	100.00

Abbreviations: CPD, Combustible cigarettes per day; ENDS, electronic nicotine delivery systems; HnB, heat-not-burn products. Note: Data are presented as unweighted n (weighted row % (R) and column % (C)). The p-value is under 0.0001 from the Rao-Scott Chi-square test, analyzing the association between the CPD categories (CPD <10, 10≤CPD<20, 20≤CPD) and the types of ENDS use (HnB, Vape, and both ENDS) among dual users only (N = 578)

As shown in the footnote of Table 2, a statistically significant association (p < 0.0001) was found between the categories of CPD and the types of ENDS used (HnB, Vape, or both) among dual users only (n = 578). In general, the proportion of participants engaging in dual use was the highest among light smokers (CPD <10) at 26.57% (15.39% + 7.83% + 3.35%); this proportion decreased as CPD increased (17.68% for moderate smokers (10–19 CPD) and 16.00% for heavy smokers (20+ CPD), which supports the rationale for conducting stratified analyses.

### Associations between different types of smoking behavior and AR

#### Multivariable associations in the general population

Table 3 presents the multivariable survey-weighted associations between exclusive CC smoking and AR. After adjusting for potential confounders, exclusive CC-only smokers showed significantly lower odds of AR diagnosis compared to lifetime never-smokers (AOR = 0.691; 95% CI = 0.562–0.850; p < 0.001). To ensure temporal precedence, a sensitivity analysis was conducted by restricting the population to those whose CC initiation preceded or coincided with their AR diagnosis (Supplementary Table 1). In this temporally-validated subset, the inverse association for exclusive CC smokers became even more pronounced (AOR = 0.519; 95% CI = 0.415–0.648; p < 0.001).

**Table 3:** Multivariable survey-weighted association between exclusive combustible cigarettes smoking and allergic rhinitis

Characteristics	Crude OR (95% CI)	P-value	Adjusted OR (95% CI)	P-value
**Smoking type**				
Never (Ref.)				
Former	0.727∗∗∗ (0.645–0.82)	0	1.125 (0.967–1.308)	0.128
CC-only	0.471∗∗∗ (0.393–0.565)	0	0.691∗∗∗ (0.562–0.85)	0
**Gender**				
Male (Ref.)				
Female			1.375∗∗∗ (1.204–1.572)	0
**Age**				
<30 (Ref.)				
30–39			0.928 (0.753–1.144)	0.485
40–49			0.699∗∗ (0.565–0.864)	0.001
50–59			0.585∗∗∗ (0.457–0.75)	0
60–69			0.408∗∗∗ (0.311–0.536)	0
70+			0.28∗∗∗ (0.202–0.388)	0
**Marital status**				
Single (Ref.)				
Married			0.956 (0.777–1.176)	0.671
Divorced/Widowed/Separated			1.055 (0.811–1.373)	0.691
**Occupation**				
Unemployed (Ref.)				
Managers, professionals, office workers			0.945 (0.827–1.079)	0.402
Sales, service workers			0.911 (0.773–1.075)	0.271
Agriculture, forestry, Fisheries			0.602∗ (0.399–0.91)	0.016
Trades workers, laborers			0.781∗∗ (0.668–0.915)	0.002
**Education**				
≤Middle school (Ref.)				
High school			1.38∗∗ (1.138–1.673)	0.001
≥College			1.41∗∗ (1.144–1.739)	0.001
**Income**				
Quintile 1 (Ref.)				
Quintile 2			1.005 (0.8–1.263)	0.966
Quintile 3			1.01 (0.812–1.258)	0.927
Quintile 4			1.086 (0.866–1.363)	0.473
Quintile 5			1.066 (0.85–1.338)	0.579
**Drinking status**				
Non-drinker (Ref.)				
Moderate drinker			0.987 (0.863–1.128)	0.845
Heavy drinker			0.817∗ (0.696–0.959)	0.013
**Weight status**				
Normal (Ref.)				
Underweight			0.946 (0.754–1.187)	0.631
Pre-obesity			1.007 (0.887–1.144)	0.914
Obesity			0.985 (0.875–1.109)	0.799
**Stress level**				
Almost none (Ref.)				
Moderate			1.113 (0.952–1.302)	0.178
High			1.492∗∗∗ (1.251–1.779)	0
**Residence in apartment housing**				
No (Ref.)				
Yes			1.05 (0.936–1.177)	0.407
**Living with children under 13**				
No (Ref.)				
Yes			1.114 (0.944–1.315)	0.2
**Investigation year**				
2019 (Ref.)				
2020	1.043 (0.893–1.219)	0.593	1.028 (0.884–1.196)	0.722
2021	0.975 (0.833–1.142)	0.756	0.991 (0.848–1.158)	0.907
2022	1.128 (0.953–1.337)	0.162	1.18 (0.997–1.397)	0.054

∗∗∗ for p < 0.001, ∗∗ for 0.001 <= p < 0.01, ∗ for 0.01 <= p < 0.05.

Notably, a strong socioeconomic gradient was observed; individuals with higher educational attainment (≥College: AOR = 1.41; 95% CI = 1.144–1.739; p = 0.001) had significantly higher odds of diagnosis. Conversely, those engaged in Agriculture, Forestry, and Fisheries (AOR = 0.602; 95% CI = 0.399–0.910; p = 0.016) showed markedly lower odds of AR compared to the unemployed.

Table 4 summarizes the associations among individuals with no history of CC smoking. In this group, exclusive ENDS-only users did not show a statistically significant difference in AR diagnosis odds compared to never-smokers (AOR = 1.174; p = 0.805). Interestingly, the protective association of older age was most pronounced here, with the oldest group (age 70+) showing 75% lower odds of AR diagnosis (AOR = 0.253; p < 0.001) compared to those under 30.

**Table 4:** Multivariable survey-weighted association between exclusive ENDS use and allergic rhinitis

Characteristics	Crude OR (95% CI)	P-value	Adjusted OR (95% CI)	P-value
**Smoking type**				
Never (Ref.)				
Former	0.883 (0.301–2.588)	0.82	0.641 (0.213–1.928)	0.428
ENDS-only	1.598 (0.455–5.614)	0.464	1.174 (0.327–4.22)	0.805
**Gender**				
Male (Ref.)				
Female			1.226∗ (1.047–1.435)	0.011
**Age**				
<30 (Ref.)				
30–39			0.914 (0.727–1.151)	0.445
40–49			0.716∗∗ (0.557–0.919)	0.009
50–59			0.588∗∗∗ (0.439–0.787)	0
60–69			0.452∗∗∗ (0.328–0.623)	0
70+			0.253∗∗∗ (0.166–0.385)	0
**Marital status**				
Single (Ref.)				
Married			0.941 (0.741–1.196)	0.62
Divorced/Widowed/Separated			1.108 (0.804–1.526)	0.531
**Occupation**				
Unemployed (Ref.)				
Managers, professionals, office workers			0.952 (0.821–1.102)	0.507
Sales, service workers			0.903 (0.742–1.098)	0.307
Agriculture, forestry, Fisheries			0.575 (0.292–1.131)	0.109
Trades workers, laborers			0.632∗∗∗ (0.508–0.785)	0
**Education**				
≤Middle school (Ref.)				
High school			1.437∗∗ (1.118–1.848)	0.005
≥College			1.529∗∗ (1.174–1.991)	0.002
**Income**				
Quintile 1 (Ref.)				
Quintile 2			0.913 (0.687–1.213)	0.528
Quintile 3			0.903 (0.694–1.174)	0.445
Quintile 4			0.984 (0.752–1.287)	0.905
Quintile 5			0.894 (0.682–1.172)	0.417
**Drinking status**				
Non-drinker (Ref.)				
Moderate drinker			0.986 (0.847–1.148)	0.858
Heavy drinker			0.754∗∗ (0.619–0.918)	0.005
**Weight status**				
Normal (Ref.)				
Underweight			0.966 (0.755–1.235)	0.782
Pre-obesity			0.946 (0.807–1.108)	0.487
Obesity			0.921 (0.801–1.059)	0.248
**Stress level**				
Almost none (Ref.)				
Moderate			1.156 (0.956–1.397)	0.135
High			1.502∗∗∗ (1.212–1.861)	0
**Residence in apartment housing**				
No (Ref.)				
Yes			1.042 (0.905–1.2)	0.568
**Living with children under 13**				
No (Ref.)				
Yes			1.141 (0.948–1.373)	0.163
**Investigation year**				
2019 (Ref.)				
2020	1.011 (0.837–1.22)	0.91	1.003 (0.835–1.206)	0.972
2021	0.937 (0.78–1.126)	0.489	0.953 (0.794–1.144)	0.606
2022	1.172 (0.962–1.427)	0.114	1.202 (0.987–1.464)	0.067

∗∗∗ for p < 0.001, ∗∗ for 0.001 <= p < 0.01, ∗ for 0.01 <= p < 0.05.

The comprehensive analysis in Table 5 confirmed that exclusive CC-only smokers had significantly lower odds of AR (AOR = 0.841; p = 0.046), while former smokers were associated with 19% higher odds (AOR = 1.193; p = 0.013). However, the sensitivity analysis restricted to those with established temporal precedence revealed a different pattern (Supplementary Table 2). In this subset, exclusive CC-only smokers (AOR = 0.520; p < 0.001), exclusive ENDS-only users (AOR = 0.588; p = 0.004), and dual users (AOR = 0.628; p = 0.007) all exhibited significantly lower odds of AR diagnosis compared to never-smokers.

**Table 5:** Multivariable survey-weighted association between comprehensive smoking patterns and allergic rhinitis

Characteristics	Crude OR (95% CI)	P-value	Adjusted OR (95% CI)	P-value
**Smoking type**				
Never (Ref.)				
Former	0.806∗∗∗ (0.722–0.9)	0	1.193∗ (1.039–1.369)	0.013
CC-only	0.632∗∗∗ (0.545–0.733)	0	0.841∗ (0.709–0.997)	0.046
ENDS-only	1.064 (0.805–1.406)	0.664	1.06 (0.792–1.419)	0.695
CC × ENDS	1.226 (0.97–1.549)	0.087	1.199 (0.93–1.546)	0.162
**Gender**				
Male (Ref.)				
Female			1.372∗∗∗ (1.22–1.542)	0
**Age**				
<30 (Ref.)				
30–39			0.846 (0.706–1.014)	0.071
40–49			0.622∗∗∗ (0.514–0.752)	0
50–59			0.498∗∗∗ (0.4–0.621)	0
60–69			0.353∗∗∗ (0.276–0.451)	0
70+			0.238∗∗∗ (0.175–0.323)	0
**Marital status**				
Single (Ref.)				
Married			1.015 (0.847–1.215)	0.873
Divorced/Widowed/Separated			1.168 (0.924–1.476)	0.194
**Occupation**				
Unemployed (Ref.)				
Managers, professionals, Office workers			0.929 (0.822–1.05)	0.24
Sales, service workers			0.967 (0.832–1.124)	0.662
Agriculture, forestry, Fisheries			0.583∗∗ (0.389–0.873)	0.009
Trades workers, laborers			0.759∗∗∗ (0.654–0.881)	0
**Education**				
≤Middle school (Ref.)				
High school			1.342∗∗ (1.11–1.623)	0.002
≥College			1.4∗∗ (1.144–1.713)	0.001
**Income**				
Quintile 1 (Ref.)				
Quintile 2			1 (0.81–1.235)	0.997
Quintile 3			0.985 (0.801–1.212)	0.889
Quintile 4			1.049 (0.848–1.297)	0.658
Quintile 5			1.051 (0.849–1.301)	0.648
**Drinking status**				
Non-drinker (Ref.)				
Moderate drinker			0.99 (0.866–1.133)	0.888
Heavy drinker			0.842∗ (0.725–0.978)	0.025
**Weight status**				
Normal (Ref.)				
Underweight			0.93 (0.749–1.156)	0.513
Pre-obesity			1.014 (0.901–1.141)	0.821
Obesity			0.962 (0.863–1.072)	0.479
**Stress level**				
Almost none (Ref.)				
Moderate			1.081 (0.929–1.258)	0.312
High			1.44∗∗∗ (1.218–1.703)	0
**Residence in apartment Housing**				
No (Ref.)				
Yes			1.027 (0.925–1.141)	0.617
**Living with children under 13**				
No (Ref.)				
Yes			1.078 (0.926–1.255)	0.332
**Investigation year**				
2019 (Ref.)				
2020	1.044 (0.906–1.203)	0.553	1.03 (0.897–1.182)	0.679
2021	1.041 (0.899–1.206)	0.588	1.064 (0.921–1.229)	0.401
2022	1.18∗ (1.016–1.37)	0.03	1.237∗∗ (1.064–1.437)	0.006

∗∗∗ for p < 0.001, ∗∗ for 0.001 <= p < 0.01, ∗ for 0.01 <= p < 0.05.

Furthermore, the positive association for former smokers observed in the primary model was reversed in this validated subgroup (AOR = 0.836; 95% CI = 0.721–0.970; p = 0.018), suggesting that the primary findings for former smokers may have been influenced by individuals who quit smoking after their diagnosis. Across all models, high stress levels consistently emerged as a strong correlate, with those in the high-stress group showing approximately 1.4–1.5 times higher odds of AR diagnosis (Table 5: AOR = 1.440; p < 0.001).

#### Stratified associations among current smokers according to smoking intensity

**Fig. 2:**
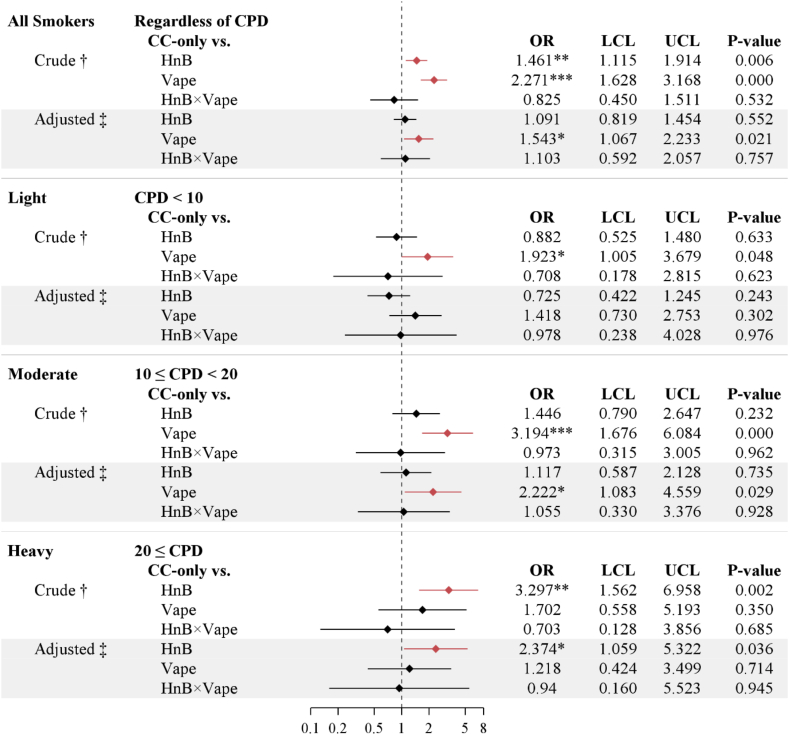
Odds ratios for allergic rhinitis by cigarettes per day (CPD): Comparison of Former, CC-only, heated tobacco product (HnB), vaping, and their Interaction (HnB × Vape) in the crude and adjusted models. Note: Horizontal axis is on a logarithmic scale. † Adjusted for investigation year only; ‡ Adjusted for sex, age, marital status, occupation, education, income, drinking status, weight status, stress level, residence in apartment housing, living with children under 13 years, and investigation year. ∗∗∗ for p < 0.001, ∗∗ for 0.001 <= p < 0.01, ∗ for 0.01 <= p < 0.05

Fig. 2 illustrates the risk patterns for AR among current smokers, stratified by CC smoking intensity, through a forest plot.

In the overall analysis of current smokers regardless of CPD, the dual use of CC and vapes was associated with significantly higher odds of AR diagnosis (AOR = 1.543; 95% CI = 1.067–2.233; p = 0.021), whereas the association for CC and HnB dual use did not reach statistical significance (AOR = 1.091; p = 0.552). Subgroup analyses by smoking intensity revealed that while light smokers (<10 CPD) showed no statistically significant associations for any dual-use patterns after adjusting for confounders, moderate smokers (10–19 CPD) who used both CC and vapes exhibited a more than two-fold increase in the odds of AR diagnosis compared to exclusive CC smoking (AOR = 2.222; 95% CI = 1.083–4.559; p = 0.029). For heavy smokers (≥20 CPD), the dual use of CC and HnB was the only pattern significantly associated with increased odds of AR diagnosis (AOR = 2.374; 95% CI = 1.059–5.322; p = 0.036). Detailed results of the full models, including all demographic and lifestyle covariates for these specific sub-populations, are provided in Supplementary Table 3–6.

## Discussion

In this study, we analyzed the associations between various smoking behaviors and the odds of allergic rhinitis (AR) using KNHANES data from 2019 to 2022. Our findings, validated through sensitivity analyses to establish temporal precedence, yielded 3 pivotal insights.

First, exclusive CC-only smokers showed significantly lower odds of AR diagnosis compared to lifetime never-smokers (AOR = 0.841, p = 0.046), a protective-style association that became even more pronounced after addressing potential reverse causality (AOR = 0.520, p < 0.001). Second, while the primary model initially showed higher odds for former smokers, our temporally-validated analysis reversed this direction (AOR = 0.836, p = 0.018), suggesting that the initial positive association was likely an artifact of individuals quitting smoking following an AR diagnosis. Third, the dual-user group demonstrated a significantly higher risk of AR in specific intensity-stratified subgroups (eg, AOR = 2.374 for heavy CC smokers who also use HnB), suggesting a synergistic risk that may override nicotine-mediated suppression.

### Socio-demographic trends and the diagnostic gradient

The socio-demographic trends observed in our study are largely consistent with established epidemiological patterns in South Korea and other industrialized nations. Our finding that AR prevalence significantly decreases with age is supported by large-scale population studies using KNHANES data, which also identified male sex and high stress levels as prominent risk factors.^13^^,^^14^ Specifically, the 1.4–1.5-fold increase in AR odds among our high-stress group aligns with the reported association between psychological stress and allergic outcomes.^14^

A notable finding was the "socioeconomic gradient," where individuals with higher education (≤College: AOR = 1.4 to 1.529) and those in professional or office-based occupations exhibited significantly higher odds of AR diagnosis. This pattern is consistent with recent findings in European populations, where higher levels of education and tertiary occupation were associated with increased AR risk.^15^ Conversely, our observation that farmers and laborers showed lower odds (AOR = 0.383 to 0.627) mirrors previous Korean data where these specific occupational groups were at lower risk of diagnosis.^14^

While these results might suggest higher true prevalence among higher Socioeconomic Status (SES) groups, they necessitate a critical evaluation of "diagnostic bias." Historically, low SES was associated with a reduced risk of AR. However, recent cohort data indicate that the role of social class has evolved, with the steepest increases in AR now occurring in populations with low SES.^16^ Thus, the higher diagnosis odds among highly educated individuals in our sample likely reflect differential healthcare utilization; those at higher occupational levels are more likely to seek and receive formal physician diagnoses.^15^ This is further driven by disparities in health literacy, as awareness of the specific term "allergic rhinitis" is often concentrated in higher socioeconomic classes, whereas lower SEC groups frequently misattribute symptoms to common colds, leading to decreased health-seeking behavior.^17^ By incorporating these SES factors as covariates, we attempted to isolate the independent associations of smoking behaviors.

### Temporal precedence and the multifaceted masking effect

Our analysis revealed a lower prevalence of AR among current CC smokers, which is potentially consistent with the hypothesis that nicotine exerts a potent anti-inflammatory effect. AR is characterized by an IgE-mediated Th2-type T cell response, and previous studies suggest that nicotine inhalation can down-modulate Th2 immunity and reduce aeroallergen sensitization.^18^, ^19^, ^20^ This immunosuppression may occur via the α7 nicotinic acetylcholine receptor (α7 nAChR) pathway^21^ or through the reduction of proinflammatory cytokines like Interleukin-33.^22^ Such pharmacological action, combined with histopathological changes in the nasal mucosa and a lower sensitivity to airborne allergens, may lead to a "masking effect" where symptoms are sufficiently attenuated to prevent patients from seeking a formal diagnosis^21^^,^^23^, ^24^, ^25^. The "sick quitter" effect was objectively demonstrated as the AOR for former smokers shifted from 1.193 (p = 0.013) to 0.836 (p = 0.018) when the temporal sequence was established. The persistence of lower odds after cessation suggests that smoking induces long-lasting immunometabolic perturbations and systemic molecular signatures that do not immediately revert.^26^

### Irritation potential of ENDS-only use

For exclusive ENDS users, no statistically significant difference in OR was observed compared to non-smokers in the primary model. However, ENDS aerosols containing propylene glycol (PG), vegetable glycerin (VG), and various flavorings can irritate the nasal mucosa and cause excessive mucus secretion 27, 28, 29. Furthermore, HnB products heat tobacco to produce harmful substances like formaldehyde and ammonia, sometimes in concentrations higher than those in CC.^30^ These components might counteract the anti-inflammatory effects of nicotine, leading to a neutral association for exclusive ENDS users.

### Synergistic risk and barrier dysfunction in dual users

Although dual users represent a minority of the total smoking population, their unique risk profile warrants heavy emphasis.^9^^,^^10^ Dual use is not merely a combination of behaviors but may create a unique, synergistic risk for AR.^31^ Simultaneous exposure to CC toxins (tar, carbon monoxide) and ENDS components (nickel, carbonyl compounds) alters ciliated cell function, reduces beat frequency, and increases inflammatory markers.^32^, ^33^, ^34^

Moreover, dual use increases oxidative stress in lung cells, weakening antioxidant defenses.^35^ This synergistic risk, irrespective of smoking quantity, may be driven by the localized disruption of the nasal epithelial barrier. While nicotine may suppress symptoms, chemical flavorings like acetoin and maltol independently induce cytokine release (eg, IL-8) and impair barrier function.^36^ Concurrent exposure could lead to the over-activation of TRPA1 receptors, downregulating essential tight junction proteins like ZO-1 and Occludin, thereby increasing mucosal permeability to aeroallergens and overriding any nicotine-mediated masking.^25^^,^^37^ In our subgroup analysis, this risk was particularly pronounced in moderate CC smokers using vapes (AOR = 2.222) and heavy CC smokers using HnB (AOR = 2.374).^38^, ^39^, ^40^, ^41^

The specific mechanisms of dual-use synergy may also involve the interaction between liquid aerosols and solid smoke particles. For instance, the combination of propylene glycol and vegetable glycerin in ENDS aerosols has been shown to induce airway inflammation and mucus hyperconcentration.^32^ When these aerosols are inhaled alongside the oxidative stressors of combustible cigarettes, they may weaken the respiratory system's antioxidant defense mechanisms more effectively than either product used alone.^35^ This cumulative chemical burden likely explains why even heavy smokers, who might otherwise experience nicotine-mediated suppression, show significantly elevated odds of AR when utilizing heated tobacco products or vapes.

### Strengths and limitations

This study is notable for utilizing a nationally representative dataset (KNHANES) with detailed smoking categorizations.^11^ Our sensitivity analysis effectively addressed reverse causality for CC users. However, several limitations remain. First, self-reported data may be subject to recall or social desirability bias. Second, the cross-sectional design makes causal relationships difficult to establish, particularly for ENDS, where initiation age was unavailable. Third, objective biomarkers are unavailable as KNHANES conducts allergen testing only in 10-year cycles. We utilized the full 2019–2022 dataset to maintain sufficient statistical power for stratified analyses; restricting the sample to the 2019 subsample would have reduced our data by over 75%, compromising the robustness of sub-group findings. Fourth, as AR prevalence was common (17.77%), the reported AORs may overestimate the true Prevalence Ratios (PRs). Future long-term prospective studies with objective biological markers like serum IgE or nasal cytology are necessary to validate these hypothetical immunologic pathways.^2^^,^^8^

## Conclusion

Our study reveals a complex and multifaceted association between smoking patterns and allergic rhinitis (AR). We confirmed that exclusive combustible cigarettes (CC) smoking is associated with significantly lower odds of AR diagnosis, a finding interpreted as an artifact of nicotine-mediated immunosuppression and symptom masking rather than a genuine protective effect. This interpretation is strongly supported by our sensitivity analysis, which addressed the "sick quitter" effect by revealing that former smokers also exhibit lower diagnosis odds once the temporal precedence of smoking initiation is established.

However, the most critical implication for public health lies in the significant synergistic risk observed among dual users. Regardless of baseline smoking intensity, the addition of electronic nicotine delivery systems (ENDS) to a CC habit consistently increased the odds of AR. Combined exposure to combustible toxins and ENDS flavorings—such as acetoin and maltol—may exert a "double-hit" on the respiratory system, inducing rapid epithelial barrier dysfunction and over-activating TRPA1 sensory receptors.

Consequently, dual use is a unique risk factor that exacerbates upper airway inflammation beyond the sum of its individual components. Clinical practitioners should be alert to more severe or persistent symptoms in dual users, and future tobacco control policies must prioritize preventing dual-use patterns to mitigate the rising burden of allergic respiratory diseases in the modern era.

## Author contributions

Dong Ju YOON: Conceptualization, Writing – original draft. Jiyoon YEO: Conceptualization, Methodology, Formal analysis, Visualization, Writing – original draft. Ye-Seul LEE: Writing – original draft, Writing – review & editing, Validation. Chunhoo CHEON: Writing – review & editing, Validation. Yoon Jae LEE: Methodology, Writing – review & editing.In-Hyuk HA: Conceptualization, Supervision, Project administration.

## Ethics statement

This study was conducted using publicly available data (KNHANES) provided by the Korea Disease Control and Prevention Agency (KDCA), and no personally identifiable information was used. In accordance with institutional regulations, the study protocol was exempted from review by the Institutional Review Board (IRB) of Jaseng Hospital of Korean Medicine (IRB approval number: 2024-09-013).

## Submission declaration

We confirm that this work is original and has not been published elsewhere, nor is it currently under consideration for publication elsewhere.

## Use of generative artificial intelligence

Nothing to disclose.

## Funding

This research received no external funding.

## Declaration of competing interest

The authors declare no conflict of interest.

## References

[1] Dierick B.J.H., van der Molen T., Flokstra-de Blok B.M.J. (2020). Burden and socioeconomics of asthma, allergic rhinitis, atopic dermatitis and food allergy. Expert Rev Pharmacoecon Outcomes Res.

[2] Savoure M., Bousquet J., Jaakkola J.J.K., Jaakkola M.S., Jacquemin B., Nadif R. (2022). Worldwide prevalence of rhinitis in adults: a review of definitions and temporal evolution. Clin Transl Allergy.

[3] Koinis-Mitchell D., Craig T., Esteban C.A., Klein R.B. (2012). Sleep and allergic disease: a summary of the literature and future directions for research. J Allergy Clin Immunol.

[4] Shargorodsky J., Garcia-Esquinas E., Galan I., Navas-Acien A., Lin S.Y. (2015). Allergic sensitization, rhinitis and tobacco smoke exposure in US adults. PLoS One.

[5] Yao T.C., Chang S.W., Chang W.C. (2017). Exposure to tobacco smoke and childhood rhinitis: a population-based study. Sci Rep.

[6] Wang S., Qi L., Wei H., Jiang F., Yan A. (2022). Smoking behavior might affect allergic rhinitis and vasomotor rhinitis differently: a mendelian randomization appraisal. World Allergy Organ J.

[7] Cherng S.T., Tam J., Christine P.J., Meza R. (2016). Modeling the effects of E-cigarettes on smoking behavior: implications for future adult smoking prevalence. Epidemiology.

[8] (2016). WHO: Electronic Nicotine Delivery Systems and Electronic Non-nicotine Delivery Systems (ENDS/ENNDS).

[9] Osibogun O., Bursac Z., Maziak W. (2022). Longitudinal transition outcomes among adult dual users of e-cigarettes and cigarettes with the intention to quit in the United States: PATH Study (2013-2018). Prev Med Rep.

[10] Coleman S.R.M., Piper M.E., Byron M.J., Bold K.W. (2022). Dual use of combustible cigarettes and E-cigarettes: a narrative review of Current evidence. Curr Addict Rep.

[11] Cheon C., Jang B.-H., Ko S.-G. (2023). A review of major secondary data resources used for research in traditional Korean medicine. Perspectives on Integrative Medicine.

[12] Lee G.N., Koo H.Y.R., Han K., Lee Y.B. (2022). Analysis of quality of life and mental health in patients with atopic dermatitis, asthma and allergic rhinitis using a nation-wide database, KNHANES VII. Allergy Asthma Immunol Res.

[13] Rhee C.S., Wee J.H., Ahn J.C. (2014). Prevalence, risk factors and comorbidities of allergic rhinitis in South Korea: the Fifth Korea National Health and Nutrition Examination Survey. Am J Rhinol Allergy.

[14] An S.Y., Choi H.G., Kim S.W. (2015). Analysis of various risk factors predisposing subjects to allergic rhinitis. Asian Pac J Allergy Immunol.

[15] Bashir M.B.A., Pullerits T., Ekerljung L. (2024). Socioeconomic status and different forms of rhinitis in Swedish adults. Clin Transl Allergy.

[16] Bråbäck L., Hjern A., Rasmussen F. (2005). Social class in asthma and allergic rhinitis: a national cohort study over three decades. Eur Respir J.

[17] Navarro-Locsin C.G., Romualdez J.A. (2016). Attitudes, practices on allergic rhinitis of three socioeconomic classes of Filipinos in the National Capital Region. Asia Pacific Allergy.

[18] Linneberg A., Nielsen N.H., Madsen F., Frolund L., Dirksen A., Jorgensen T. (2001). Smoking and the development of allergic sensitization to aeroallergens in adults: a prospective population-based study. The Copenhagen allergy study. Allergy.

[19] Mishra N.C., Rir-Sima-Ah J., Langley R.J. (2008). Nicotine primarily suppresses lung Th2 but not goblet cell and muscle cell responses to allergens. J Immunol.

[20] Nishimura T., Kaminuma O., Saeki M., Kitamura N., Mori A., Hiroi T. (2020). Suppressive effect of environmental tobacco smoke on murine Th2 cell-mediated nasal eosinophilic inflammation. Asia Pac Allergy.

[21] Hollenhorst M.I., Krasteva-Christ G. (2021). Nicotinic acetylcholine receptors in the respiratory tract. Molecules.

[22] Gomez R.M., Croce V.H., Zernotti M.E., Muino J.C. (2021). Active smoking effect in allergic rhinitis. World Allergy Organ J.

[23] Eriksson J., Ekerljung L., Sundblad B.M. (2013). Cigarette smoking is associated with high prevalence of chronic rhinitis and low prevalence of allergic rhinitis in men. Allergy.

[24] Hadar T., Yaniv E., Shvili Y., Koren R., Shvero J. (2009). Histopathological changes of the nasal mucosa induced by smoking. Inhal Toxicol.

[25] Mizobata K., Sakatani H., Kono M., Saika S., Akaike A., Hotomi M. (2026). The impact of nicotine on the olfactory memory and its relationship with TRPA1. iScience.

[26] Ma J., Liu Y., Xu H. (2026). Plasma proteomic profiling with machine learning identifies immunometabolic perturbations associated with smoking and smoking cessation. iScience.

[27] Gotts J.E., Jordt S.E., McConnell R., Tarran R. (2019). What are the respiratory effects of e-cigarettes?. BMJ.

[28] Rowell T.R., Tarran R. (2015). Will chronic e-cigarette use cause lung disease?. Am J Physiol Lung Cell Mol Physiol.

[29] Wieslander G., Norback D., Lindgren T. (2001). Experimental exposure to propylene glycol mist in aviation emergency training: acute ocular and respiratory effects. Occup Environ Med.

[30] Bekki K., Uchiyama S., Ohta K., Inaba Y., Nakagome H., Kunugita N. (2014). Carbonyl compounds generated from electronic cigarettes. Int J Environ Res Public Health.

[31] Pisinger C., Rasmussen S.K.B. (2022). The health effects of real-world dual use of electronic and conventional cigarettes versus the health effects of exclusive smoking of conventional cigarettes: a systematic review. Int J Environ Res Public Health.

[32] Kim M.D., Chung S., Baumlin N. (2024). The combination of propylene glycol and vegetable glycerin e-cigarette aerosols induces airway inflammation and mucus hyperconcentration. Sci Rep.

[33] Mercan S., Genc M.K. (2023). Known and unknown toxic substances in new-generation tobacco products. Addicta: The Turkish Journal on Addictions.

[34] Travis N., Knoll M., Cook S. (2023). Chemical profiles and toxicity of electronic cigarettes: an umbrella review and methodological considerations. Int J Environ Res Public Health.

[35] Stokes A.C., Xie W., Wilson A.E. (2021). Association of cigarette and electronic cigarette use patterns with levels of inflammatory and oxidative stress biomarkers among US adults: population assessment of tobacco and health Study. Circulation.

[36] Gerloff J., Sundar I.K., Freter R. (2017). Inflammatory response and barrier dysfunction by different e-Cigarette flavoring chemicals identified by gas chromatography–mass spectrometry in e-Liquids and e-Vapors on human lung epithelial cells and fibroblasts. Applied In Vitro Toxicology.

[37] Sun Y-b, Liu M., Fan X-s (2021). Effects of cigarette smoke on the aggravation of ovalbumin-induced asthma and the expressions of TRPA1 and tight junctions in mice. Mol Immunol.

[38] Rebuli M.E., Glista-Baker E., Hoffman J.R. (2021). Electronic-Cigarette use alters Nasal mucosal immune response to live-attenuated influenza virus. A clinical trial. Am J Respir Cell Mol Biol.

[39] Xie W., Kathuria H., Galiatsatos P. (2020). Association of electronic cigarette use with incident respiratory conditions among US adults from 2013 to 2018. JAMA Netw Open.

[40] Yamamoto T., Sekine Y., Sohara K., Nakai S., Yanagisawa Y. (2022). Effect of heating temperature on ammonia emission in the mainstream aerosols from heated tobacco products. Toxics.

[41] Zervas E.N., Matsouki N.E., Tsipa C.F., Katsaounou P.A. (2024). Particle emissions from heated tobacco products. Tobacco Prevention & Cessation.

