# Supplementary material for: Associations between dual use of combustible cigarettes and electronic nicotine delivery systems and allergic rhinitis: An analysis of KNHANES data
Source: World Allergy Organ J. 2026 Jul 2;19(8):101423. doi: 10.1016/j.waojou.2026.101423 (PMC13352082; doi:10.1016/j.waojou.2026.101423)
Supplement: Multimedia component 1 [file mmc1.docx]

Supplementary Table 1. Sensitivity analysis for the association between exclusive combustible cigarette smoking and allergic rhinitis, restricted to individuals with tobacco initiation prior to or concurrent with AR diagnosis

| **Characteristics** | **Crude OR (95% CI)** | **P-value** | **Adjusted OR (95% CI)** | **P-value** |
| --- | --- | --- | --- | --- |
| **Smoking Type** |  |  |  |  |
| Never (Ref.) |  |  |  |  |
| Former | 0.555*** (0.488-0.631) | 0 | 0.814* (0.695-0.953) | 0.011 |
| CC-only | 0.37*** (0.303-0.452) | 0 | 0.519*** (0.415-0.648) | 0 |
| **Gender** |  |  |  |  |
| Male (Ref.) |  |  |  |  |
| Female |  |  | 1.295*** (1.129-1.485) | 0 |
| **Age** |  |  |  |  |
| <30 (Ref.) |  |  |  |  |
| 30-39 |  |  | 0.942 (0.763-1.162) | 0.576 |
| 40-49 |  |  | 0.758* (0.611-0.941) | 0.012 |
| 50-59 |  |  | 0.673** (0.523-0.867) | 0.002 |
| 60-69 |  |  | 0.484*** (0.367-0.637) | 0 |
| 70+ |  |  | 0.328*** (0.235-0.456) | 0 |
| **Marital Status** |  |  |  |  |
| Single (Ref.) |  |  |  |  |
| Married |  |  | 1.006 (0.816-1.241) | 0.953 |
| Divorced/Widowed/  Separated |  |  | 1.089 (0.833-1.424) | 0.532 |
| **Occupation** |  |  |  |  |
| Unemployed (Ref.) |  |  |  |  |
| Managers, Professionals,  Office Workers |  |  | 0.951 (0.832-1.087) | 0.459 |
| Sales, Service Workers |  |  | 0.91 (0.766-1.08) | 0.281 |
| Agriculture, Forestry,  Fisheries |  |  | 0.627* (0.414-0.949) | 0.028 |
| Trades Workers, Laborers |  |  | 0.8** (0.682-0.938) | 0.006 |
| **Education** |  |  |  |  |
| ≤Middle School (Ref.) |  |  |  |  |
| High School |  |  | 1.356** (1.114-1.651) | 0.002 |
| ≥College |  |  | 1.414** (1.139-1.756) | 0.002 |
| **Income** |  |  |  |  |
| Quintile 1 (Ref.) |  |  |  |  |
| Quintile 2 |  |  | 0.987 (0.781-1.248) | 0.913 |
| Quintile 3 |  |  | 0.994 (0.795-1.242) | 0.958 |
| Quintile 4 |  |  | 1.084 (0.861-1.364) | 0.492 |
| Quintile 5 |  |  | 1.058 (0.839-1.332) | 0.634 |
| **Drinking Status** |  |  |  |  |
| Non-Drinker (Ref.) |  |  |  |  |
| Moderate Drinker |  |  | 0.985 (0.861-1.128) | 0.829 |
| Heavy Drinker |  |  | 0.842* (0.716-0.991) | 0.038 |
| **Weight status** |  |  |  |  |
| Normal (Ref.) |  |  |  |  |
| Underweight |  |  | 0.992 (0.791-1.245) | 0.946 |
| Pre-obesity |  |  | 0.964 (0.845-1.1) | 0.588 |
| Obesity |  |  | 0.94 (0.833-1.06) | 0.314 |
| **Stress Level** |  |  |  |  |
| Almost None (Ref.) |  |  |  |  |
| Moderate |  |  | 1.128 (0.961-1.324) | 0.142 |
| High |  |  | 1.477*** (1.233-1.769) | 0 |
| **Residence in**  **Apartment Housing** |  |  |  |  |
| No (Ref.) |  |  |  |  |
| Yes |  |  | 1.043 (0.927-1.173) | 0.482 |
| **Living with**  **Children Under 13** |  |  |  |  |
| No (Ref.) |  |  |  |  |
| Yes |  |  | 1.126 (0.952-1.333) | 0.165 |
| **Investigation Year** |  |  |  |  |
| 2019 (Ref.) |  |  |  |  |
| 2020 | 1.027 (0.873-1.207) | 0.749 | 1.019 (0.871-1.194) | 0.81 |
| 2021 | 0.966 (0.821-1.137) | 0.681 | 0.986 (0.839-1.157) | 0.859 |
| 2022 | 1.127 (0.948-1.34) | 0.175 | 1.175 (0.99-1.394) | 0.065 |
| Note: This sensitivity analysis was conducted on a total of 18,430 participants to establish temporal precedence. We excluded 351 former or current smokers who initiated tobacco use after their AR diagnosis (initiation age > AR diagnosis age). The final analytical sample consisted of 7,891 smokers whose initiation occurred prior to or concurrent with their diagnosis (initiation age ≤ AR diagnosis age) and 10,536 lifetime never-smokers. | | | | |

**Supplementary Table 2.** Sensitivity analysis for the association between comprehensive smoking patterns and allergic rhinitis, restricted to individuals with tobacco initiation prior to or concurrent with AR diagnosis

| **Characteristics** | **Crude OR (95% CI)** | **P-value** | **Adjusted OR (95% CI)** | **P-value** |
| --- | --- | --- | --- | --- |
| **Smoking Type** |  |  |  |  |
| Never (Ref.) |  |  |  |  |
| Former | 0.594*** (0.527-0.669) | 0 | 0.836* (0.721-0.97) | 0.018 |
| CC-only | 0.4*** (0.335-0.478) | 0 | 0.52*** (0.427-0.634) | 0 |
| ENDS-only | 0.579** (0.409-0.819) | 0.002 | 0.588** (0.41-0.842) | 0.004 |
| CC×ENDS | 0.611** (0.443-0.843) | 0.003 | 0.628** (0.447-0.882) | 0.007 |
|  |  |  |  |  |
| **Gender** |  |  |  |  |
| Male (Ref.) |  |  |  |  |
| Female |  |  | 1.289*** (1.134-1.466) | 0 |
| **Age** |  |  |  |  |
| <30 (Ref.) |  |  |  |  |
| 30-39 |  |  | 0.936 (0.768-1.139) | 0.507 |
| 40-49 |  |  | 0.759** (0.617-0.933) | 0.009 |
| 50-59 |  |  | 0.646*** (0.511-0.818) | 0 |
| 60-69 |  |  | 0.463*** (0.358-0.6) | 0 |
| 70+ |  |  | 0.301*** (0.218-0.414) | 0 |
| **Marital Status** |  |  |  |  |
| Single (Ref.) |  |  |  |  |
| Married |  |  | 1.116 (0.92-1.353) | 0.264 |
| Divorced/Widowed/  Separated |  |  | 1.279 (0.995-1.645) | 0.055 |
| **Occupation** |  |  |  |  |
| Unemployed (Ref.) |  |  |  |  |
| Managers, Professionals,  Office Workers |  |  | 0.946 (0.835-1.073) | 0.388 |
| Sales, Service Workers |  |  | 0.933 (0.796-1.094) | 0.393 |
| Agriculture, Forestry,  Fisheries |  |  | 0.626* (0.42-0.935) | 0.022 |
| Trades Workers, Laborers |  |  | 0.799** (0.687-0.93) | 0.004 |
| **Education** |  |  |  |  |
| ≤Middle School (Ref.) |  |  |  |  |
| High School |  |  | 1.311** (1.076-1.598) | 0.007 |
| ≥College |  |  | 1.406** (1.135-1.741) | 0.002 |
| **Income** |  |  |  |  |
| Quintile 1 (Ref.) |  |  |  |  |
| Quintile 2 |  |  | 0.969 (0.774-1.213) | 0.785 |
| Quintile 3 |  |  | 0.935 (0.753-1.161) | 0.541 |
| Quintile 4 |  |  | 1.011 (0.813-1.259) | 0.92 |
| Quintile 5 |  |  | 1.016 (0.813-1.27) | 0.888 |
| **Drinking Status** |  |  |  |  |
| Non-Drinker (Ref.) |  |  |  |  |
| Moderate Drinker |  |  | 0.977 (0.852-1.119) | 0.734 |
| Heavy Drinker |  |  | 0.823* (0.701-0.967) | 0.018 |
| **Weight status** |  |  |  |  |
| Normal (Ref.) |  |  |  |  |
| Underweight |  |  | 1.014 (0.812-1.268) | 0.901 |
| Pre-obesity |  |  | 0.956 (0.843-1.085) | 0.488 |
| Obesity |  |  | 0.915 (0.816-1.027) | 0.13 |
| **Stress Level** |  |  |  |  |
| Almost None (Ref.) |  |  |  |  |
| Moderate |  |  | 1.157 (0.989-1.354) | 0.069 |
| High |  |  | 1.55*** (1.3-1.848) | 0 |
| **Residence in**  **Apartment Housing** |  |  |  |  |
| No (Ref.) |  |  |  |  |
| Yes |  |  | 1.046 (0.938-1.166) | 0.416 |
| **Living with**  **Children Under 13** |  |  |  |  |
| No (Ref.) |  |  |  |  |
| Yes |  |  | 1.106 (0.945-1.295) | 0.211 |
| **Investigation Year** |  |  |  |  |
| 2019 (Ref.) |  |  |  |  |
| 2020 | 1.033 (0.886-1.205) | 0.675 | 1.033 (0.891-1.199) | 0.664 |
| 2021 | 0.984 (0.843-1.149) | 0.842 | 1.014 (0.871-1.181) | 0.856 |
| 2022 | 1.158 (0.989-1.356) | 0.068 | 1.216* (1.04-1.421) | 0.014 |
| Note: This sensitivity analysis was conducted on a total of 18,430 participants to establish temporal precedence. We excluded 351 former or current smokers who initiated tobacco use after their AR diagnosis (initiation age > AR diagnosis age). The final analytical sample consisted of 7,891 smokers whose initiation occurred prior to or concurrent with their diagnosis (initiation age ≤ AR diagnosis age) and 10,536 lifetime never-smokers. | | | | |

Supplementary Table 3. Multivariable survey-weighted logistic regression analysis for allergic rhinitis among total current smokers

| **Characteristics** | **Crude OR (95% CI)** | **P-value** | **Adjusted OR (95% CI)** | **P-value** |
| --- | --- | --- | --- | --- |
| **Smoking Type** |  |  |  |  |
| CC-only (Ref.) |  |  |  |  |
| HnB | 1.461** (1.115-1.914) | 0.006 | 1.091 (0.819-1.454) | 0.552 |
| Vaping | 2.271*** (1.628-3.168) | 0 | 1.543* (1.067-2.233) | 0.021 |
| HnB×Vaping | 0.825 (0.45-1.511) | 0.532 | 1.103 (0.592-2.057) | 0.757 |
| **Gender** |  |  |  |  |
| Male (Ref.) |  |  |  |  |
| Female |  |  | 1.592** (1.208-2.1) | 0.001 |
| **Age** |  |  |  |  |
| <30 (Ref.) |  |  |  |  |
| 30-39 |  |  | 0.738 (0.512-1.065) | 0.105 |
| 40-49 |  |  | 0.47*** (0.323-0.683) | 0 |
| 50-59 |  |  | 0.368*** (0.235-0.574) | 0 |
| 60-69 |  |  | 0.178*** (0.102-0.312) | 0 |
| 70+ |  |  | 0.068*** (0.027-0.172) | 0 |
| **Marital Status** |  |  |  |  |
| Single (Ref.) |  |  |  |  |
| Married |  |  | 1.281 (0.889-1.846) | 0.184 |
| Divorced/Widowed/  Separated |  |  | 1.923** (1.224-3.024) | 0.005 |
| **Occupation** |  |  |  |  |
| Unemployed (Ref.) |  |  |  |  |
| Managers, Professionals,  Office Workers |  |  | 0.936 (0.676-1.296) | 0.69 |
| Sales, Service Workers |  |  | 1.206 (0.861-1.689) | 0.275 |
| Agriculture, Forestry,  Fisheries |  |  | 0.712 (0.319-1.59) | 0.407 |
| Trades Workers, Laborers |  |  | 0.807 (0.568-1.146) | 0.23 |
| **Education** |  |  |  |  |
| ≤Middle School (Ref.) |  |  |  |  |
| High School |  |  | 1.185 (0.75-1.87) | 0.467 |
| ≥College |  |  | 1.239 (0.778-1.975) | 0.366 |
| **Income** |  |  |  |  |
| Quintile 1 (Ref.) |  |  |  |  |
| Quintile 2 |  |  | 1.391 (0.822-2.353) | 0.218 |
| Quintile 3 |  |  | 1.028 (0.616-1.716) | 0.916 |
| Quintile 4 |  |  | 1.055 (0.624-1.783) | 0.843 |
| Quintile 5 |  |  | 1.402 (0.838-2.344) | 0.198 |
| **Drinking Status** |  |  |  |  |
| Non-Drinker (Ref.) |  |  |  |  |
| Moderate Drinker |  |  | 1.021 (0.659-1.581) | 0.925 |
| Heavy Drinker |  |  | 1.004 (0.663-1.522) | 0.983 |
| **Weight status** |  |  |  |  |
| Normal (Ref.) |  |  |  |  |
| Underweight |  |  | 0.857 (0.453-1.623) | 0.636 |
| Pre-obesity |  |  | 1.068 (0.793-1.437) | 0.665 |
| Obesity |  |  | 0.904 (0.694-1.178) | 0.455 |
| **Stress Level** |  |  |  |  |
| Almost None (Ref.) |  |  |  |  |
| Moderate |  |  | 0.976 (0.658-1.447) | 0.902 |
| High |  |  | 1.452 (0.986-2.137) | 0.059 |
| **Residence in**  **Apartment Housing** |  |  |  |  |
| No (Ref.) |  |  |  |  |
| Yes |  |  | 0.936 (0.745-1.177) | 0.571 |
| **Living with**  **Children Under 13** |  |  |  |  |
| No (Ref.) |  |  |  |  |
| Yes |  |  | 0.839 (0.605-1.164) | 0.293 |
| **Investigation Year** |  |  |  |  |
| 2019 (Ref.) |  |  |  |  |
| 2020 | 1.1 (0.839-1.442) | 0.492 | 1.073 (0.812-1.418) | 0.619 |
| 2021 | 1.169 (0.873-1.565) | 0.294 | 1.174 (0.876-1.572) | 0.283 |
| 2022 | 1.247 (0.919-1.691) | 0.156 | 1.343 (0.983-1.836) | 0.064 |

Supplementary Table 4. Multivariable survey-weighted logistic regression analysis for allergic rhinitis among light smokers (<10 cigarettes per day)

| **Characteristics** | **Crude OR (95% CI)** | **P-value** | **Adjusted OR (95% CI)** | **P-value** |
| --- | --- | --- | --- | --- |
| **Smoking Type** |  |  |  |  |
| CC-only (Ref.) |  |  |  |  |
| HnB | 0.882 (0.525-1.48) | 0.633 | 0.725 (0.422-1.245) | 0.243 |
| Vaping | 1.923* (1.005-3.679) | 0.048 | 1.418 (0.73-2.753) | 0.302 |
| HnB×Vaping | 0.708 (0.178-2.815) | 0.623 | 0.978 (0.238-4.028) | 0.976 |
| **Gender** |  |  |  |  |
| Male (Ref.) |  |  |  |  |
| Female |  |  | 1.613* (1.04-2.501) | 0.033 |
| **Age** |  |  |  |  |
| <30 (Ref.) |  |  |  |  |
| 30-39 |  |  | 0.979 (0.49-1.955) | 0.952 |
| 40-49 |  |  | 0.664 (0.325-1.354) | 0.259 |
| 50-59 |  |  | 0.725 (0.296-1.779) | 0.482 |
| 60-69 |  |  | 0.358 (0.123-1.045) | 0.06 |
| 70+ |  |  | 0.096** (0.02-0.46) | 0.003 |
| **Marital Status** |  |  |  |  |
| Single (Ref.) |  |  |  |  |
| Married |  |  | 1.043 (0.528-2.059) | 0.904 |
| Divorced/Widowed/  Separated |  |  | 0.907 (0.362-2.276) | 0.835 |
| **Occupation** |  |  |  |  |
| Unemployed (Ref.) |  |  |  |  |
| Managers, Professionals,  Office Workers |  |  | 0.685 (0.385-1.22) | 0.198 |
| Sales, Service Workers |  |  | 1.104 (0.619-1.968) | 0.738 |
| Agriculture, Forestry,  Fisheries |  |  | 0.834 (0.187-3.71) | 0.811 |
| Trades Workers, Laborers |  |  | 0.761 (0.408-1.418) | 0.389 |
| **Education** |  |  |  |  |
| ≤Middle School (Ref.) |  |  |  |  |
| High School |  |  | 0.942 (0.407-2.178) | 0.888 |
| ≥College |  |  | 0.922 (0.394-2.156) | 0.851 |
| **Income** |  |  |  |  |
| Quintile 1 (Ref.) |  |  |  |  |
| Quintile 2 |  |  | 1.672 (0.692-4.039) | 0.253 |
| Quintile 3 |  |  | 1.304 (0.584-2.915) | 0.516 |
| Quintile 4 |  |  | 1.163 (0.499-2.715) | 0.726 |
| Quintile 5 |  |  | 1.497 (0.645-3.475) | 0.347 |
| **Drinking Status** |  |  |  |  |
| Non-Drinker (Ref.) |  |  |  |  |
| Moderate Drinker |  |  | 0.694 (0.307-1.57) | 0.38 |
| Heavy Drinker |  |  | 0.872 (0.382-1.99) | 0.744 |
| **Weight status** |  |  |  |  |
| Normal (Ref.) |  |  |  |  |
| Underweight |  |  | 0.885 (0.337-2.323) | 0.803 |
| Pre-obesity |  |  | 1.002 (0.582-1.724) | 0.995 |
| Obesity |  |  | 0.949 (0.597-1.51) | 0.826 |
| **Stress Level** |  |  |  |  |
| Almost None (Ref.) |  |  |  |  |
| Moderate |  |  | 1.124 (0.573-2.206) | 0.733 |
| High |  |  | 1.295 (0.628-2.667) | 0.483 |
| **Residence in**  **Apartment Housing** |  |  |  |  |
| No (Ref.) |  |  |  |  |
| Yes |  |  | 0.817 (0.524-1.273) | 0.371 |
| **Living with**  **Children Under 13** |  |  |  |  |
| No (Ref.) |  |  |  |  |
| Yes |  |  | 0.971 (0.538-1.753) | 0.923 |
| **Investigation Year** |  |  |  |  |
| 2019 (Ref.) |  |  |  |  |
| 2020 | 1.048 (0.617-1.779) | 0.863 | 0.944 (0.56-1.59) | 0.827 |
| 2021 | 1.193 (0.66-2.157) | 0.559 | 1.117 (0.635-1.963) | 0.701 |
| 2022 | 1.413 (0.772-2.585) | 0.262 | 1.437 (0.763-2.705) | 0.261 |

Supplementary Table 5. Multivariable survey-weighted logistic regression analysis for allergic rhinitis among moderate smokers (10–19 cigarettes per day)

| **Characteristics** | **Crude OR (95% CI)** | **P-value** | **Adjusted OR (95% CI)** | **P-value** |
| --- | --- | --- | --- | --- |
| **Smoking Type** |  |  |  |  |
| CC-only (Ref.) |  |  |  |  |
| HnB | 1.446 (0.79-2.647) | 0.232 | 1.117 (0.587-2.128) | 0.735 |
| Vaping | 3.194*** (1.676-6.084) | 0 | 2.222* (1.083-4.559) | 0.029 |
| HnB×Vaping | 0.973 (0.315-3.005) | 0.962 | 1.055 (0.33-3.376) | 0.928 |
| **Gender** |  |  |  |  |
| Male (Ref.) |  |  |  |  |
| Female |  |  | 1.733* (1.049-2.863) | 0.032 |
| **Age** |  |  |  |  |
| <30 (Ref.) |  |  |  |  |
| 30-39 |  |  | 0.704 (0.395-1.256) | 0.235 |
| 40-49 |  |  | 0.473* (0.254-0.883) | 0.019 |
| 50-59 |  |  | 0.326** (0.156-0.682) | 0.003 |
| 60-69 |  |  | 0.117*** (0.046-0.297) | 0 |
| 70+ |  |  | 0.07*** (0.016-0.305) | 0 |
| **Marital Status** |  |  |  |  |
| Single (Ref.) |  |  |  |  |
| Married |  |  | 1 (0.56-1.783) | 0.999 |
| Divorced/Widowed/  Separated |  |  | 1.752 (0.812-3.781) | 0.152 |
| **Occupation** |  |  |  |  |
| Unemployed (Ref.) |  |  |  |  |
| Managers, Professionals,  Office Workers |  |  | 1.512 (0.819-2.793) | 0.186 |
| Sales, Service Workers |  |  | 1.458 (0.79-2.692) | 0.227 |
| Agriculture, Forestry,  Fisheries |  |  | 1.914 (0.511-7.162) | 0.334 |
| Trades Workers, Laborers |  |  | 1.042 (0.571-1.901) | 0.893 |
| **Education** |  |  |  |  |
| ≤Middle School (Ref.) |  |  |  |  |
| High School |  |  | 1.215 (0.589-2.509) | 0.597 |
| ≥College |  |  | 1.149 (0.549-2.403) | 0.712 |
| **Income** |  |  |  |  |
| Quintile 1 (Ref.) |  |  |  |  |
| Quintile 2 |  |  | 0.829 (0.369-1.865) | 0.651 |
| Quintile 3 |  |  | 0.57 (0.254-1.279) | 0.172 |
| Quintile 4 |  |  | 0.796 (0.357-1.774) | 0.575 |
| Quintile 5 |  |  | 1.048 (0.464-2.363) | 0.911 |
| **Drinking Status** |  |  |  |  |
| Non-Drinker (Ref.) |  |  |  |  |
| Moderate Drinker |  |  | 1.319 (0.605-2.872) | 0.485 |
| Heavy Drinker |  |  | 1.217 (0.579-2.556) | 0.603 |
| **Weight status** |  |  |  |  |
| Normal (Ref.) |  |  |  |  |
| Underweight |  |  | 0.49 (0.147-1.636) | 0.246 |
| Pre-obesity |  |  | 0.894 (0.545-1.466) | 0.656 |
| Obesity |  |  | 0.663 (0.42-1.045) | 0.077 |
| **Stress Level** |  |  |  |  |
| Almost None (Ref.) |  |  |  |  |
| Moderate |  |  | 1.153 (0.613-2.167) | 0.658 |
| High |  |  | 1.904 (0.964-3.76) | 0.064 |
| **Residence in**  **Apartment Housing** |  |  |  |  |
| No (Ref.) |  |  |  |  |
| Yes |  |  | 0.831 (0.55-1.256) | 0.38 |
| **Living with**  **Children Under 13** |  |  |  |  |
| No (Ref.) |  |  |  |  |
| Yes |  |  | 0.911 (0.54-1.535) | 0.726 |
| **Investigation Year** |  |  |  |  |
| 2019 (Ref.) |  |  |  |  |
| 2020 |  |  | 1.342 (0.8-2.251) | 0.265 |
| 2021 |  |  | 1.333 (0.82-2.167) | 0.246 |
| 2022 |  |  | 1.737* (1.047-2.882) | 0.032 |

Supplementary Table 6. Multivariable survey-weighted logistic regression analysis for allergic rhinitis among heavy smokers (≥20 cigarettes per day)

| **Characteristics** | **Crude OR (95% CI)** | **P-value** | **Adjusted OR (95% CI)** | **P-value** |
| --- | --- | --- | --- | --- |
| **Smoking Type** |  |  |  |  |
| CC-only (Ref.) |  |  |  |  |
| HnB | 3.297** (1.562-6.958) | 0.002 | 2.374* (1.059-5.322) | 0.036 |
| Vaping | 1.702 (0.558-5.193) | 0.35 | 1.218 (0.424-3.499) | 0.714 |
| HnB×Vaping | 0.703 (0.128-3.856) | 0.685 | 0.94 (0.16-5.523) | 0.945 |
| **Gender** |  |  |  |  |
| Male (Ref.) |  |  |  |  |
| Female |  |  | 1.351 (0.495-3.691) | 0.556 |
| **Age** |  |  |  |  |
| <30 (Ref.) |  |  |  |  |
| 30-39 |  |  | 1.261 (0.423-3.753) | 0.677 |
| 40-49 |  |  | 0.651 (0.233-1.817) | 0.412 |
| 50-59 |  |  | 0.515 (0.176-1.506) | 0.225 |
| 60-69 |  |  | 0.222* (0.052-0.957) | 0.044 |
| 70+ |  |  | 0.034** (0.005-0.255) | 0.001 |
| **Marital Status** |  |  |  |  |
| Single (Ref.) |  |  |  |  |
| Married |  |  | 1.72 (0.778-3.801) | 0.179 |
| Divorced/Widowed/  Separated |  |  | 2.666* (1.078-6.593) | 0.034 |
| **Occupation** |  |  |  |  |
| Unemployed (Ref.) |  |  |  |  |
| Managers, Professionals,  Office Workers |  |  | 1.349 (0.496-3.669) | 0.557 |
| Sales, Service Workers |  |  | 1.565 (0.543-4.509) | 0.406 |
| Agriculture, Forestry,  Fisheries |  |  | 0.294 (0.052-1.661) | 0.165 |
| Trades Workers, Laborers |  |  | 1.126 (0.438-2.894) | 0.805 |
| **Education** |  |  |  |  |
| ≤Middle School (Ref.) |  |  |  |  |
| High School |  |  | 0.899 (0.392-2.066) | 0.802 |
| ≥College |  |  | 1.35 (0.563-3.239) | 0.501 |
| **Income** |  |  |  |  |
| Quintile 1 (Ref.) |  |  |  |  |
| Quintile 2 |  |  | 19.578** (2.971-129.004) | 0.002 |
| Quintile 3 |  |  | 9.296* (1.335-64.707) | 0.024 |
| Quintile 4 |  |  | 5.402 (0.788-37.044) | 0.086 |
| Quintile 5 |  |  | 10.376* (1.561-68.966) | 0.016 |
| **Drinking Status** |  |  |  |  |
| Non-Drinker (Ref.) |  |  |  |  |
| Moderate Drinker |  |  | 0.928 (0.378-2.278) | 0.87 |
| Heavy Drinker |  |  | 0.854 (0.379-1.922) | 0.702 |
| **Weight status** |  |  |  |  |
| Normal (Ref.) |  |  |  |  |
| Underweight |  |  | 2.439 (0.399-14.908) | 0.334 |
| Pre-obesity |  |  | 1.967 (0.885-4.374) | 0.097 |
| Obesity |  |  | 1.393 (0.669-2.903) | 0.375 |
| **Stress Level** |  |  |  |  |
| Almost None (Ref.) |  |  |  |  |
| Moderate |  |  | 0.805 (0.302-2.145) | 0.663 |
| High |  |  | 1.073 (0.394-2.924) | 0.89 |
| **Residence in**  **Apartment Housing** |  |  |  |  |
| No (Ref.) |  |  |  |  |
| Yes |  |  | 1.243 (0.684-2.258) | 0.475 |
| **Living with**  **Children Under 13** |  |  |  |  |
| No (Ref.) |  |  |  |  |
| Yes |  |  | 0.407* (0.185-0.897) | 0.026 |
| **Investigation Year** |  |  |  |  |
| 2019 (Ref.) |  |  |  |  |
| 2020 | 0.885 (0.402-1.949) | 0.761 | 1.018 (0.453-2.283) | 0.966 |
| 2021 | 1.518 (0.744-3.097) | 0.25 | 1.73 (0.826-3.622) | 0.146 |
| 2022 | 0.92 (0.428-1.979) | 0.83 | 1.155 (0.522-2.556) | 0.721 |
